# Editorial: Current trends in environmental psychology, volume I

**DOI:** 10.3389/fpsyg.2023.1228180

**Published:** 2023-07-03

**Authors:** Eugenio De Gregorio, Federica Caffaro, Sabine Pirchio, Lorenza Tiberio, Giuseppe Carrus

**Affiliations:** ^1^Department of Life and Health Science, Link Campus University, Rome, Lazio, Italy; ^2^Department of Education, Roma Tre University, Rome, Lazio, Italy; ^3^Department of Dynamic and Clinical Psychology and Health Studies, Sapienza University of Rome, Rome, Lazio, Italy

**Keywords:** environmental psychology, restorative environments, human wellbeing, climate change perceptions and adaptation, ecological behaviors, environmental education, transitions to sustainability

This Research Topic on “*Current trends in environmental psychology, volume I*”, which is linked to the 3rd International Conference of Environmental Psychology (ICEP 2021), held in Siracusa, Italy, 4–9 October 2021, constituted an enormous challenge. Our proposal was aimed to promote an interdisciplinary turnaround in environmental psychological thinking, at a time when major existential challenges posed by the raise of new technologies in everyday life settings (homes, workplaces, hospitals, schools, green areas, urban open spaces), and by the worsening of the global emergency at the planetary level, seem to be at their peak.

The still ongoing transformations of human social life induced by the COVID-19 pandemic, and in general different ways of interacting and communicating are bringing about significant changes in terms of models and methodologies in different areas of psychological science, including environmental psychology.

Through the call for papers of this RT, we wanted to reconstruct and debate the new research trends within the field of environmental psychology. Beyond the specific fields of application covered in the papers included in this RT, this experience has shown us how broad the scientific community is within the disciplinary context of environmental psychology, in terms of methods, theories and contents of investigation.

Foundational matters have been re-evaluated and brought up to date, such as experimental research on the perception and representation of spaces, the effects of heat on aggression, and the psychological basis of sustainable behavioral choices. For example, in this Research Topic, studies have been included attempting to identify the possible rationale in human behavior regarding environmental protection in different contexts (urban and rural, coastal and mountain). All these are classic themes of environmental psychological research.

Some articles have also taken up other classic themes of environmental psychology, reinterpreting them in a modern key and referring to theories that seemed to have been forgotten, perhaps because linked to a deterministic perspective that gradually became less popular in present-day psychology and in particular in environmental psychology.

Significant relevance is devoted to the challenges posed by people's public transport choices (air and road) also in connection with the recent COVID-19 pandemic. In the pandemic and post-pandemic era, the issue of sustainable mobility intersects with that of health protection, with implications on the concept of 'all-round health' and planetary wellbeing. This aspect also makes it relevant to keep a closer look at policies and decision-making strategies for the promotion of social and environmental sustainability, for the conservation of biodiversity and protection of animal and vegetal species, to ensure the optimal functioning of earth's ecosystems.

An important application context, which is also covered in this RT, is that of educational settings, considered from the perspective of environmental psychology as spaces of action and thought where the values and strategies and actions of future generations are forged.

The pandemic issue is present in many papers and not only because of the problems it has brought to human life in the last 3 years, but also because of the value of discovering resources and people's sense of social adaptation: a tragedy, certainly, but also an adaptation resource that guided the discovery of new potential in human societies.

From a methodological point of view, many different approaches and research traditions are represented in this RT. We included studies that have made use of complex experimental designs and large scale surveys, as well as theoretical contributions and studies with a more general epistemological inspiration. Also, importantly, we included studies that have made use of qualitative methods.

Researchers from the Americas, Europe (in particular, Germany, Belgium and the Netherlands) and China have contributed to the co-construction of a collective scientific endeavor that at this moment has collected 58,000 views across 34 different papers.

## Conclusion

In conclusion, it is also interesting to point out potential areas of improvement in present day environmental psychological research, namely the fact that an explicit, integrated and comprehensive methodological framework in environmental psychology (for example through new trends such as mixed methods) still needs to be defined and constructed by empirical research in our field, as represented by the various papers included in this RT.

## Author contributions

All authors contributed equally to writing and revising the text of the editorial and to conceptualizing and editing the Research Topic.

